# *De novo* donor-specific HLA antibody development after kidney transplantation is impacted by PIRCHE II score and recipient age

**DOI:** 10.3389/fimmu.2025.1508586

**Published:** 2025-04-01

**Authors:** Yuan Tian, Lukas Frischknecht, Fabian Rössler, Thomas Schachtner, Jakob Nilsson

**Affiliations:** ^1^ Department of Immunology, University Hospital Zurich (USZ), Zurich, Switzerland; ^2^ Department of Surgery and Transplantation, University Hospital Zurich, Zurich, Switzerland; ^3^ Division of Nephrology, University Hospital Zurich, Zurich, Switzerland

**Keywords:** kidney transplantation, PIRCHE, donor specific antibodies, virtual cross-match, age

## Abstract

**Background:**

Antibody-mediated rejection (ABMR) is a major cause of graft loss in kidney transplantation, often associated with *de novo* donor-specific antibodies (dnDSA). The detection of clinically relevant dnDSA relies on evaluating reactivity in single antigen bead (SAB) assays. Immunogenetic mismatches between donor and recipient, particularly involving human leukocyte antigens (HLA), underpin dnDSA development. Understanding this relationship could improve pre-transplant risk assessment and organ allocation.

**Methods:**

We analyzed 1296 kidney transplant patients to study dnDSA development, its relation to age, gender, and the role of HLA-derived peptide mismatches using the Predicted Indirectly Recognizable HLA Epitopes II (PIRCHE II) score. We categorized dnDSA based on bead reactivity patterns and HLA typing into true, possible, and false dnDSA.

**Results:**

During follow-up, 25% of recipients developed dnDSA, 9.3% true, 7.7% possible, and 7.9% false. True dnDSA primarily targeted HLA-DQ (38%), while HLA-C and HLA-DP were uncommon (5% and 3%). Higher PIRCHE II scores were significantly associated with true and possible dnDSA against HLA Class II compared to false dnDSA, supporting our dnDSA classification. For true and possible dnDSA, the single locus PIRCHE II score strongly correlated with locus-specific dnDSA, while the total PIRCHE II score did not appear to influence locus-specific dnDSA development. Younger recipients exhibited a higher risk of dnDSA development, while gender had no impact.

**Conclusion:**

Locus-specific PIRCHE II scores are useful in predicting dnDSA risk post-transplantation, particularly in younger recipients. Promoting transplants with low PIRCHE II scores against key HLA loci like HLA-DQ in younger recipients could improve outcomes.

## Introduction

Antibody mediated rejection (ABMR) remains the dominant cause of graft loss in the setting of kidney transplantation (1). Donor human leukocyte antigen (HLA) protein variants that are mismatched to the recipient are key targets for this T-cell dependent alloimmune response. The appearance of *de novo* donor specific antibodies (dnDSA) or the presence of pre-transplant DSA targeting donor HLA proteins is tightly associated with the development of ABMR (2–4). This association often results in accelerated graft loss and necessitates the reinitiation of dialysis. Furthermore, it may lead to the need for a second kidney transplantation. The intensified or additional immunosuppression used to treat ABMR is also associated with increased morbidity and mortality predominantly due to infections (5). A subsequent transplantation following a previous graft loss due to ABMR is often complicated by a broad immunization against foreign HLA protein variants. This results in a high calculated panel reactive antibody (cPRA), which impairs the possibility of finding a suitable donor (6). Taken together, this highlights the importance of avoiding dnDSA development and subsequent ABMR in the effort to improve the long-term outcome of kidney transplantation. The development of dnDSA is highly dependent on the HLA mismatch between the donor and recipient where each subsequent mismatch has been shown to be associated with an increased risk of graft loss (7). However, not all HLA mismatches are immunologically equivalent, as they are dependent on the current HLA nomenclature and do not account for the immunogenicity of the mismatch in a specific donor recipient pair. Predicted indirectly recognizable HLA Epitopes II (PIRCHE II) is a method for predicting the number of donor HLA derived peptides that can be presented to the recipients T cells on the recipient Class II HLA proteins (8). The PIRCHE II score has been shown to provide a more detailed estimation of the risk associated with individual HLA mismatches and has, together with other HLA epitope focused tools such as the HLA MatchMaker, led to an improvement in pre-transplant immunogenetical risk prediction (9). Previous studies have shown that the PIRCHE II score is tightly associated with the development of dnDSA after kidney transplantation (10–12). Nonetheless, these studies rely heavily on the specificity of the detected dnDSA where there are numerous issues related to unspecific results (13). A thorough assessment of the detected dnDSAs is necessary, considering allele or alpha chain dependency, as well as assessment of unspecific reactivity. Unspecific reactivity, or detected dnDSAs that are not truly donor specific would be predicted to influence the estimation of the PIRCHE II score association with dnDSA development. In order to further investigate these important transplant immunological questions, we have performed a careful evaluation of dnDSA development in a cohort of 1296 patients who received a kidney transplant at the University Hospital in Zurich between January 2008 and March 2024.

## Methods

### Patient population

The patients included in this study underwent kidney transplantation between January 2008 and March 2024 at the University Hospital of Zürich (USZ). Only patients that were monitored for the development of dnDSA at the USZ post-transplant were included into the study (approximately 90% of transplanted patients). The dataset comprises complete information from a total of 1296 patients (808 males and 488 females), and the recorded data encompass sex, date of birth, date of transplantation, HLA-type of the donor and recipient, PIRCHE II score (HLA-A, B, C, DRB1, DQ), post-transplant follow-up time, as well as data on dnDSA development. The study was approved by the local Ethical Committee in Zurich (BASEC 2018-01182).

### HLA typing and anti-HLA antibody analysis

DNA based HLA typing was conducted using blood samples, employing either sequence-specific oligonucleotide (SSO), sequence-specific primer (SSP) or Next generation sequencing (NGS) technologies. In addition to the standard donor HLA typing, further typing was performed to assess additional loci if the recipient developed anti-HLA antibodies after transplantation against an HLA locus that had not been preciously typed. Therefore, a complete virtual cross-match (vXM) for the post-transplant dnDSA development was available for all patients included in the study.

HLA antibody screening was performed after transplantation according to the local protocol at the USZ for post-transplant follow-up and conducted by use of Luminex single-antigen bead (SAB) technology (LABScreen Single Antigen; OneLambda). Monitoring for dnDSA was performed in accordance with the protocol at 1, 3, 6, 12, 18 and 24 months after transplantation and then on a yearly basis thereafter. Additional anti-HLA antibody testing was also performed in the setting of clinical suspicion of rejection at the discretion of the treating physician. Positivity of dnDSAs were defined by the presence of dnDSA targeting the HLA loci A, B, C, DR (including DRB3,4 and 5), DQ and DP with a normalized mean fluorescence intensity (MFI) exceeding 500.

### *De novo* DSA evaluation

The dnDSA that were detected post-transplant by use of our automated vXM algorithm, which evaluates donor-recipient compatibility in silico based on recipient anti-HLA antibodies (measured via Single Antigen Bead assays) and donor HLA molecular typing at antigen based resolution, were then analyzed individually by a specialist in transplantation immunology in a blinded fashion. Here it was determined if the antibody showed true donor specificity by analyzing the pattern of single bead reactivity and comparing it to the HLA typing of the donor. Detected dnDSA were also investigated for epitope specificity to determine alpha chain binding antibodies in the setting of HLA-DQ and DP.

Furthermore, the pattern of reactivity was compared to lot specific reactivity patterns in non-immunized males that are continuously tracked in the transplant laboratory of the USZ to allow for the determination of lot-specific unspecific reactivity. Reactivity in the SAB analysis to the recipients own HLA antigens were also incorporated into this evaluation.

Based on this analysis the dnDSA reactivity was dived into three categories as described in the results section. True dnDSA consisted of dnDSA with correct bead-based donor specificity without concerns for unspecific reactivity. Possible dnDSA included cases where true dnDSA specificity could not be definitively confirmed or excluded due to HLA typing results and the absence of a clear pattern of unspecific reactivity, yet where the reactivity did not represent clear dnDSA. False dnDSA consisted of cases where true bead-based donor specificity could be excluded, as well as individuals where a clear unspecific reactivity was noted.

Patients with pre-transplant DSA were only deemed to have developed dnDSA if a new DSA emerged post-transplant.

### Predicted indirectly recognizable HLA-Epitopes (PIRCHE II) score calculation

The number of donor-mismatched HLA-derived peptide epitopes was calculated using the PIRCHE II algorithm v3 (https://www.pirche.org). The algorithm aims to predict the presentation of donor HLA-derived peptides by the recipient’s HLA class II molecules using a machine learning-based algorithm. PIRCHE II scores were separately calculated for each HLA locus: HLA-A, HLA-B, HLA-C, HLA-DRB1, and HLA-DQB1. For class I scores, the PIRCHE II score is the sum of HLA-A, HLA-B, and HLA-C scores, while for class II scores, it is the sum of HLA-DRB1 and HLA-DQB1 scores. The total PIRCHE II score is the sum of all loci scores for each donor-recipient pair. Our calculations were restricted to peptide presentation via HLA-DRB1, based on imputed HLA types available in the PIRCHE-II algorithm. Therefore, we did not calculate scores for HLA-DP, HLA-DRB3, 4 and 5, or assess peptide presentation via HLA-DQA1, all of which could potentially influence alloimmune responses and dnDSA formation. In the setting of using aggregated locus specific scores, we considered each recipient/donor locus match as a single risk constellation. Subsequently, for several analyses, we assessed if this risk constellation was associated with the development of a dnDSA targeting this specific locus. The PIRCHE-II cut-offs (<30, 30–90, 90–150, >150) were established by assessing dnDSA incidence across various thresholds and identifying the ranges that best differentiated risk groups in our cohort.

### Statistical methods

Several statistical tests were applied to assess significance in this study. These included Kaplan-Meier analysis with the log-rank test for comparing the incidence of dnDSA between groups, T-test for unpaired data to analyze differences in PIRCHE II scores between two groups, one-way analysis of variance (ANOVA) with Bonferroni correction for comparing differences in PIRCHE II scores among multiple groups. Additionally, a Kruskal-Wallis test followed by Dunn’s *post hoc* test was used to compare the MFI values among more than two groups, due to the non-parametric nature of the data. Spearman correlations were also conducted to evaluate relationships between MFI values and PIRCHE II scores. Cox regression models were employed to assess the relative risk of developing dnDSA, calculating hazard ratios and their corresponding 95% confidence intervals (CIs). These models included the correlation between either logarithmic PIRCHE-II scores or age groups and the cumulative incidence of dnDSA. Statistical analyses were processed using R (version 4.2.4) and RStudio (version 2023.12.1 + 402). The following packages were utilized: readxl (1.4.3), dplyr (1.1.2), ggplot2 (3.4.2), swimplot (1.2.0), lubridate (1.9.3), viridis (0.6.5), webr (0.1.5), dunn.test (1.3.5), ggbeeswarm (0.7.2), multcomp (1.4.25), forcats (1.0.0), survival (3.5.5), survminer (0.4.9), gridExtra (2.3).

Statistical significance was determined based on individual p-values, with a threshold of <0.05 considered significant.

## Results

### Study population characteristics

**Figure 1:**
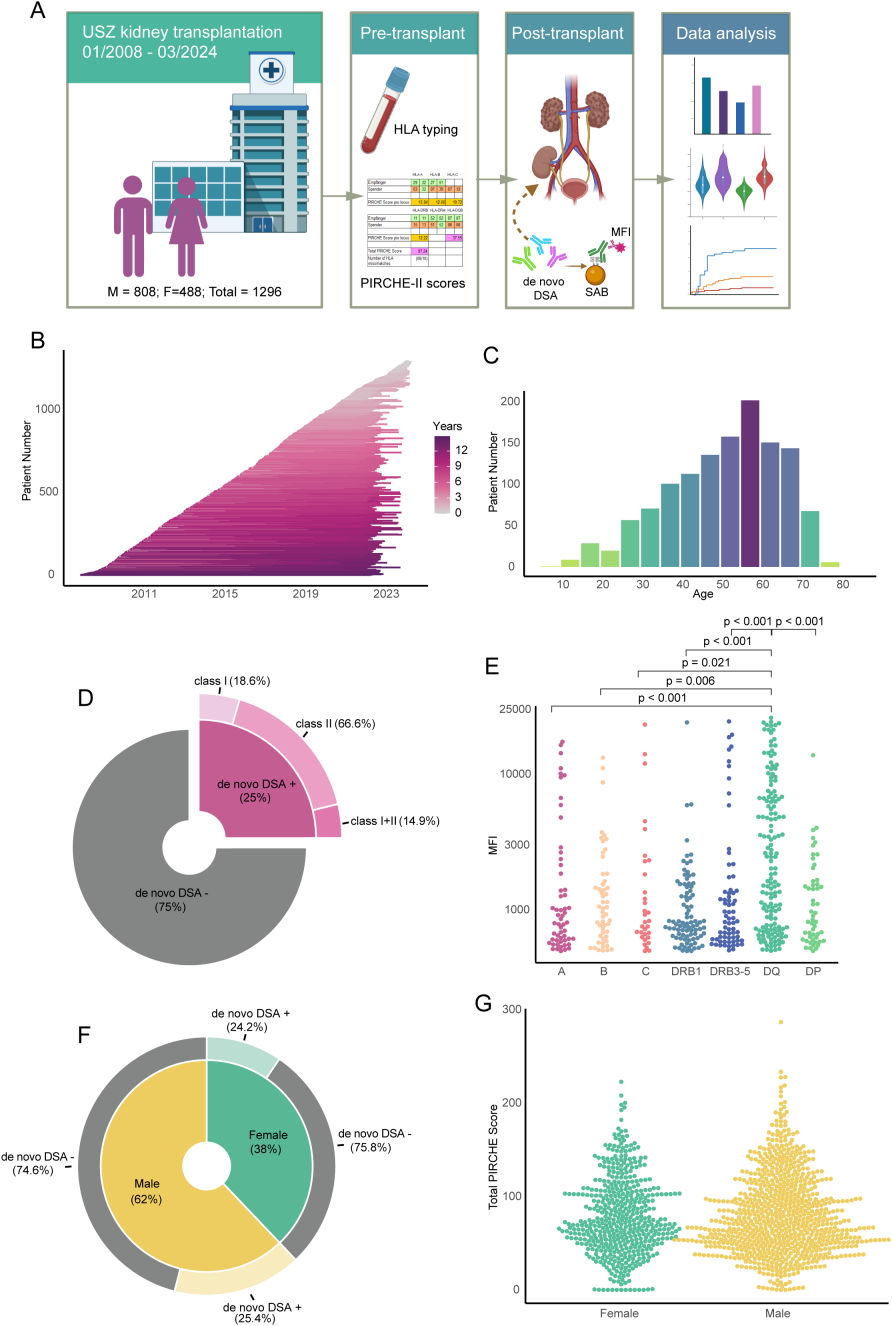
Overview of the study cohort and patient characteristics: *de novo* DSA, MFI, PIRCHE II scores and gender distribution. **(A)** Workflow of the study, illustrating patient enrolment, laboratory data collection, and major data analysis steps. **(B)** Follow-up timeline for individual patients from transplant date. **(C)** Age distribution of the total patient population. **(D)** Pie chart displaying the percentage of *de novo* DSA within the total patient population and categorization of the *de novo* DSA. **(E)** MFI values for DSA against each single HLA locus in patients with detected *de novo* DSA. **(F)** Pie chart indicating gender distribution in the study and percentage of *de novo* DSA-positive patients in each gender. **(G)** Summary of total PIRCHE II scores of each patient, grouped by gender.

An illustrative summary of patient inclusion and the data analysis processes is presented in **Figure 1A**. The study encompassed patients who received a kidney transplant at the University Hospital Zurich within the period from 2008 to 2024 and were subjected to post-transplant monitoring for the emergence of *de novo* donor-specific antibodies (dnDSA). The cohort totaled 1,296 individuals, comprising 808 male and 488 female recipients. Detailed information on the mean follow-up duration post-transplantation is delineated in **Figure 1B**. The inclusion criteria were not limited by donor type or recipient age, thereby encompassing both adult and pediatric transplantations from living and deceased donors. The distribution of recipient ages at the time of transplantation is depicted in **Figure 1C**. In total, 25% of the transplant recipients developed a dnDSA post-transplant, and the majority of the dnDSA were directed at HLA Class II (**Figure 1D**). The peak MFI distribution of the individual dnDSA showed significantly higher MFI values for those directed at HLA-DQ compared to dnDSA directed at HLA-A, B, C, DRB1, DRB3-5 and DP (**Figure 1E**). Comparative analyses focusing on the incidence of dnDSA development and the aggregate PIRCHE II scores between male and female recipients revealed no discernible differences, as shown in **Figures 1F, G**.

### *De novo* DSA evaluation

We performed a detailed evaluation of the dnDSA detected through the use of an automated virtual cross-match (vXM) algorithm, supplemented with additional HLA typing as needed to complete the vXM. In this blinded analysis, dnDSA were assessed for potential allele or alpha chain dependency, specifically in the context of HLA-DQ and DP, and for association with known unspecific reactive beads, based on the study of anti-HLA antibody reactivity in non-immunized males.

**Figure 2:**
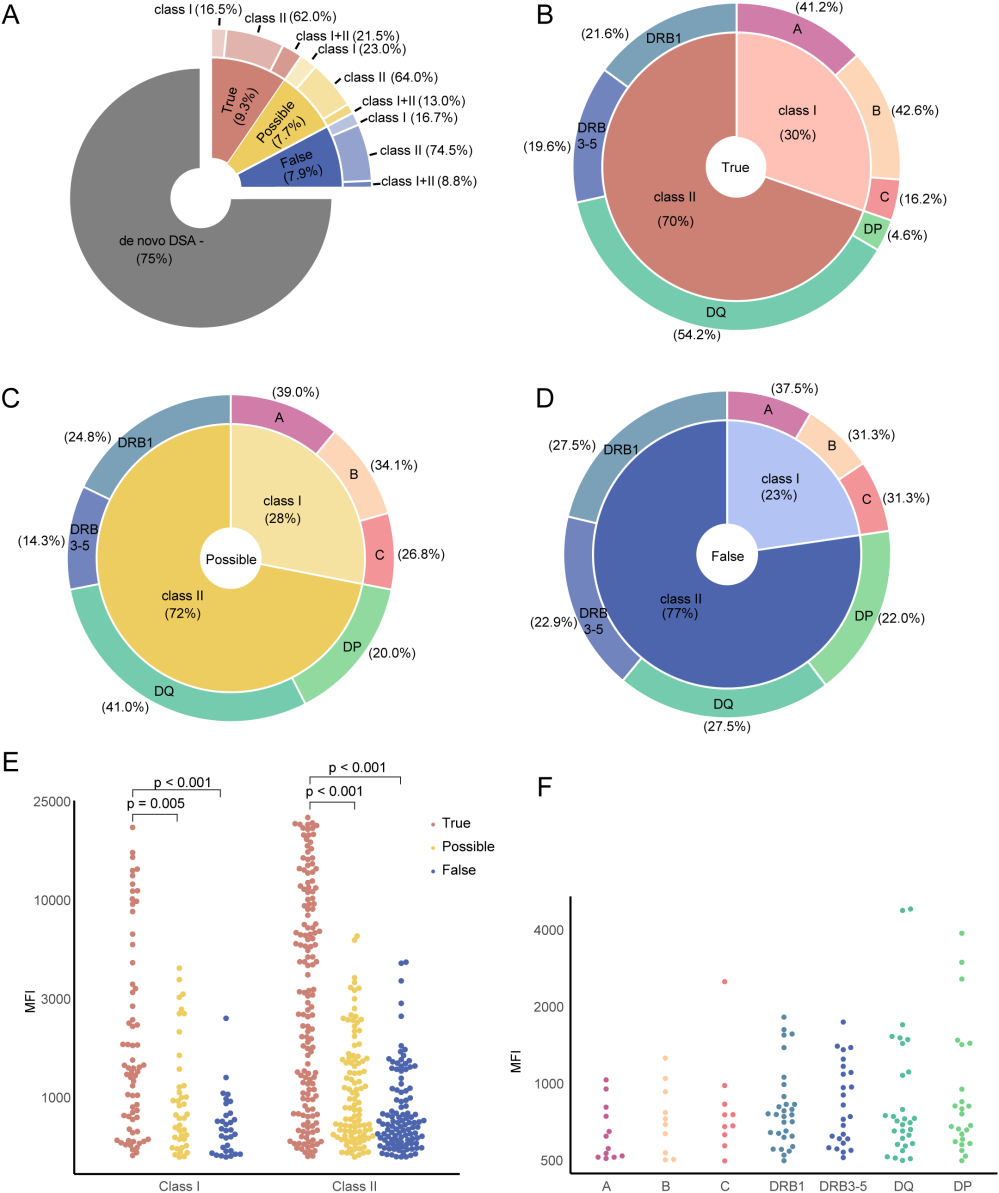
Description of *de novo* DSA annotation. **(A)** Percentage of *de novo* DSA marked into true, possible and false groups. **(B)** Distribution of true DSA types as shown in **(A)**. The inner circle represents the percentage of HLA class I and II antigen-specific dnDSAs, while the outer circle provides a detailed breakdown of the percentage of each dnDSA subtype. **(C)** Percentage of possible dnDSA types from **(A)**. **(D)** Distribution of false dnDSA types as depicted in **(A)**. **(E)** Summary of MFI values from individual patients categorized into HLA class I and II antigen-specific, and colored by the annotation. **(F)** Detail of MFI values by DSA target locus from false dnDSA group.

Following this detailed analysis, dnDSA were assessed as true in 9.3% of patients, possible in 7.7%, and false in 7.9% (**Figure 2A**). Notably, the true group contained more transplants with a combination of dnDSA directed against both Class I and Class II antigens (21.5%) compared to the possible (13.0%) and false groups (8.8%) (**Figure 2A**). We next investigated the frequency of targeted HLA loci within our three dnDSA categories (**Figures 2B–D**). Notable differences were observed, such as the high frequency of dnDSA targeting HLA-DQ in the true group (54.2%) compared to both the possible (41.0%) and the false group (27.5%). Conversely, dnDSA targeting HLA-DP were less common in the true group (4.6%), while both the possible and the false group showed higher frequencies (20.0% and 22.0%). This is likely due to the high frequency of unspecific reactivity associated with HLA-DP bearing beads and the difficulty in interpreting this reactivity. The same trend, albeit at a lower frequency, was also observed for HLA-C (**Figures 2B–D**). The dnDSA target HLA loci distribution for the whole dnDSA group can be seen in **Supplementary Figure S1**. Next, we compared the peak MFI values of the individual dnDSA within our three categories, observing a significantly higher MFI associated with the true dnDSA category compared to both the possible and false categories for both Class I and Class II directed dnDSA (**Figure 2E**). This primarily occurred because the possible and false groups had an overrepresentation of low MFI antibodies. Low MFI is typical for unspecific reactivity in the SAB assay. This can be clearly visualized in a detailed plot of the MFI values from the individual dnDSA in the false group, where the few antibodies with higher MFI values typically consisted of allele-specific or alpha chain dependent antibodies (non-DSA) (**Figure 2F**). A detailed table on dnDSA combinations within the cohort can be seen in **Supplementary Table S1**.

### PIRCHE II score is associated with *de novo* DSA development

**Figure 3:**
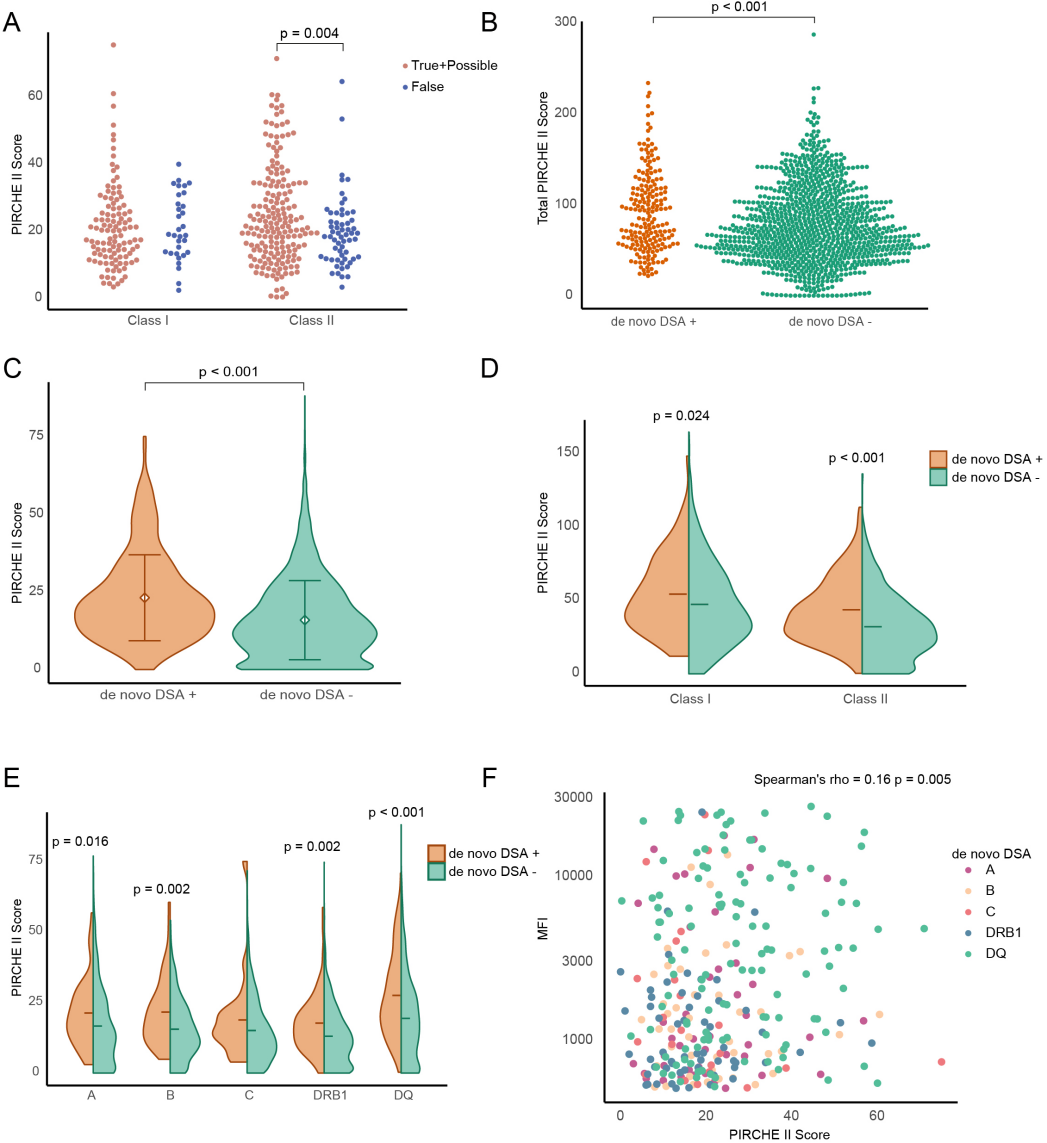
The correlation of PIRCHE II scores and MFI of *de novo* DSA in patients from true and possible groups. **(A)** Comparison of PIRCHE II scores between the combined true and possible groups and the false group in HLA class I and II antigen-specific DSA. Each dot represents one *de novo* DSA event. **(B)** Beeswarm plot showing total PIRCHE II scores form each patient. **(C)** Individual PIRCHE II locus scores in dnDSA-positive and dnDSA-negative events. **(D, E)** PIRCHE II locus scores in different HLA classes antigen-specific **(D)** and dnDSA sub-types **(E)** from **(C)**. **(F)** Scatter plot of Spearman’s rank correlation coefficient between single PIRCHE score and dnDSA MFI value.

We proceeded to examine the correlation between the PIRCHE II score and dnDSA development within our cohort. Notably, HLA-DP was excluded from all PIRCHE related analyses due to reliance on imputed high-resolution HLA types for our population, which do not encompass HLA-DP, thereby omitting dnDSA targeting HLA-DP. Additionally, dnDSA against HLA-DR were considered only if they targeted HLA-DRB1, as PIRCHE II values were not generated for the related DRB chains (DRB3, 4, and 5) in our analysis. Merging the true and possible dnDSA categories, we compared the aggregate Class I and II PIRCHE II scores with those of the false category, identifying a significant difference in PIRCHE II scores for true and possible dnDSA directed at HLA Class II antigens (**Figure 3A**). For further analyses, the true and possible dnDSA categories were amalgamated into a dnDSA positive group, while the false dnDSA category was excluded. A significant difference in total PIRCHE II scores was observed when comparing dnDSA positive patients (true and possible) to those without dnDSA (**Figure 3B**). This finding was supported by a hazard ratio significantly exceeding 1.0 in the Cox regression univariate analysis for the total PIRCHE II score on the prediction of dnDSA (**Table 1**). In order to increase the granularity of our analysis we evaluated each locus mismatch between recipient and donor as a possible risk constellation and investigated the individual locus PIRCHE II scores between positive and negative dnDSA scenarios which again reaffirmed a significant association between PIRCHE II locus score and dnDSA development (**Figure 3C**). Employing the same analytical strategy to amalgamate the PIRCHE II scores for Class I (A,B,C) and Class II (DRB1, DQ) for further examination of dnDSA positive and negative risk scenarios revealed significant differences both for Class I and II, with a trend suggesting a larger difference for Class II (**Figure 3D**). This approach was then applied to assess the impact of individual locus PIRCHE II scores in relation to dnDSA development against the same HLA locus. We found significantly elevated scores for HLA-A, B, DRB1, and DQ, but interestingly not for HLA-C (**Figure 3E**). This was also confirmed by the hazard ratios from the Cox regression univariate analysis of the PIRCHE II scores for individual HLA locus in predicting dnDSA; all hazard ratios were significantly higher than 1.0, except for HLA-C, which was not significant (**Table 1**). Lastly, a spearman analysis was conducted to explore the potential impact of individual locus PIRCHE II scores on the peak dnDSA MFI post-transplantation and this demonstrated a significant correlation, albeit with a low rho value (**Figure 3F**).

**Table 1:** Univariate cox regression model for PIRCHE II scores at total and individual HLA locus on the prediction of dnDSA (n = 1194).

Logarithmic PIRCHE II for each HLA locus univariate analysis		
	HR (95% CI)	P
Total HLA	1.80 (1.40-2.32)	<0.001
HLA-A	1.75 (1.22-2.51)	0.002
HLA-B	2.40 (1.46-3.93)	<0.001
HLA-C	1.58 (0.90-2.77)	0.112
HLA-DRB1	1.81 (1.29-2.53)	<0.001
HLA-DQ	1.98 (1.57-2.51)	<0.001

CI, confidence interval; HR, hazard ratio; PIRCHE, predicted indirectly recognizable HLA epitopes.

### PIRCHE II score and the kinetics of *de novo* DSA development

**Figure 4:**
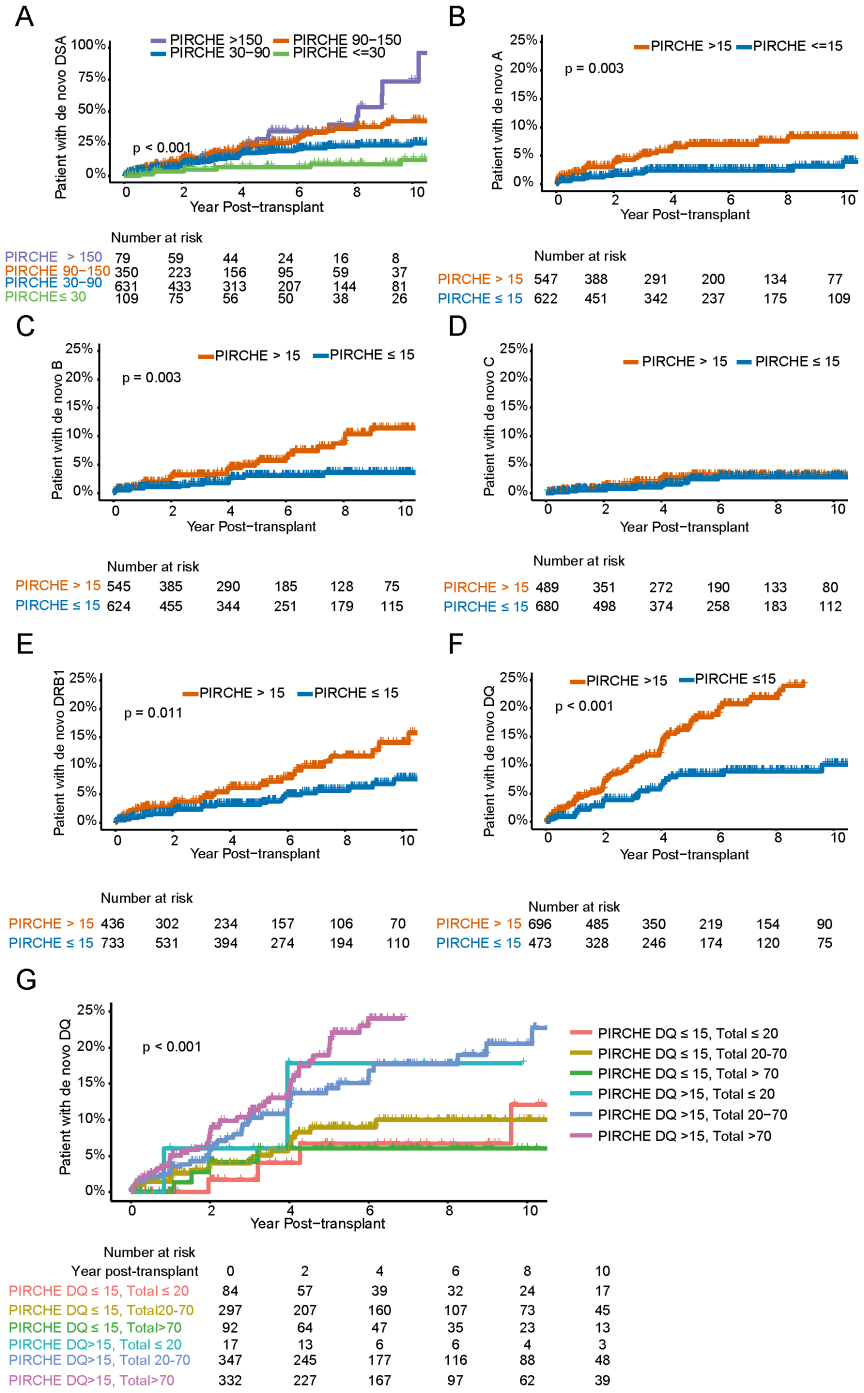
Kaplan-Meier plots showing the cumulative incidence of *de novo* DSA groups according to the PIRCHE II scores. **(A)** Stratified based on total PIRCHE II scores ≤ 30, 30-90, 90-150, > 150). **(B-F)** Cumulative incidence of dnDSA against HLA single loci (HLA-A, HLA-B, HLA-C, HLA-DRB1, HLA-DQ, respectively), grouped by the PIRCHE II score from HLA-A, B, C, DRB1, DQ ≤ 15, > 15). **(G)** Cumulative incidence of *de novo* DSA against HLA-DQ, according to DQ PIRCHE score (≤ 15, > 15) and total PIRCHE score excluding HLA-DQ PIRCHE score (≤ 20, 20-70, > 70).

We next sought to investigate if the PIRCHE II score was associated with differences in the kinetics of dnDSA development post-transplant by using Kaplan Meier analysis. We decided to divide our cohort into four risk groups based on the total PIRCHE II score. The chosen PIRCHE II score cut-offs were determined based on our own analysis, though inspired by previously published studies (10). Here, we were able to see marked differences in dnDSA development over time within our different risk groups (**Figure 4A**). Interestingly, a difference between the total PIRCHE II 90-150 and >150 groups could only be visualized late after transplantation (>9 years). But this difference was small and based on relatively few patients at risk. We next decided to perform the same analysis on dnDSA development in the single HLA locus setting where a single locus PIRCHE II score cut-off of 15 was used to define two risk groups. Our analysis showed a significantly increased risk for dnDSA development for HLA-A, B, DRB1 and DQ, but interestingly not for HLA-C where the risk appeared to be independent of PIRCHE II score (**Figures 4B-F**). The targeted HLA locus with the highest risk for dnDSA development in the setting of a locus PIRCHE II score of >15 was HLA-DQ where approximately 25% of transplant recipients had developed a dnDSA targeting HLA-DQ at 10 years post-transplant (**Figure 4F**). We next sought to tease out if there is an additive effect of the total PIRCHE II score to the risk associated with the individual HLA locus PIRCHE II score. Since dnDSA directed against HLA-DQ was most prevalent within our dnDSA positive group and also showed a strong dependency on the locus PIRCHE II score we chose to focus on this group. To investigate this question, we divided our cohort into 6 subgroups based on the total PIRCHE II score (excluding the HLA-DQ PIRCHE score) and the HLA-DQ PIRCHE score, and analyzed dnDSA development against HLA-DQ. Again, individuals with an HLA-DQ PIRCHE II score of >15 showed an increased risk of anti-HLA-DQ dnDSA development, while those with an HLA-DQ PIRCHE II score of 15 or less exhibited a lower risk even when total PIRCHE II score from the other loci (A, B, C and DRB1) exceeded 70 (**Figure 4G**).

### The risk of dnDSA development against one locus mismatch in the setting of a high or low PIRCHE II score

**Figure 5:**
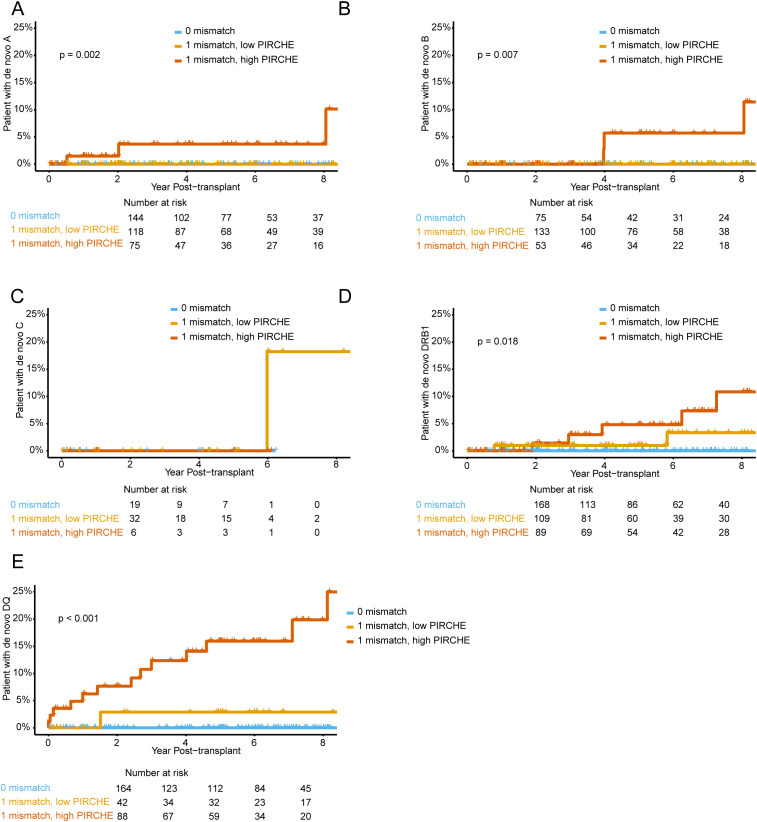
Cumulative incidence of *de novo* DSA groups according to the combinations of HLA mismatch and PIRCHE II scores. **(A-E)** Kaplan-Meier plots stratified by high (highest quartile) and low (lowest quartile) PIRCHE II scores, with 0 and 1 HLA single loci mismatch for **(A)** HLA-A, **(B)** HLA-B, **(C)** HLA-C, **(D)** HLA-DRB1 and **(E)** HLA-DQ.

In order to investigate how a high or low PIRCHE II score relates to locus specific dnDSA formation in the setting of one locus HLA mismatch we analyzed dnDSA development between the first and fourth PIRCHE II score quartiles and compared it to patients with no mismatches (**Figures 5A-E**). For HLA Class I mismatches we observed a significant difference between high and low PIRCHE II groups for HLA-A (**Figure 5A**) and for HLA-B only after 4 years post-transplantation (**Figure 5B**), but not for HLA-C (**Figure 5C**). For HLA Class II, the difference between low and high PIRCHE II scores was significant for both HLA-DRB1 and HLA-DQ (**Figures 5C, D**). The difference between the high and low PIRCHE II group was most striking for HLA-DQ, where over 20% of the individuals in the high group developed a dnDSA 8 years after transplantation, compared to only around 3% in the low group (**Figure 5E**).

### Recipient age and the kinetic of dnDSA development

**Figure 6:**
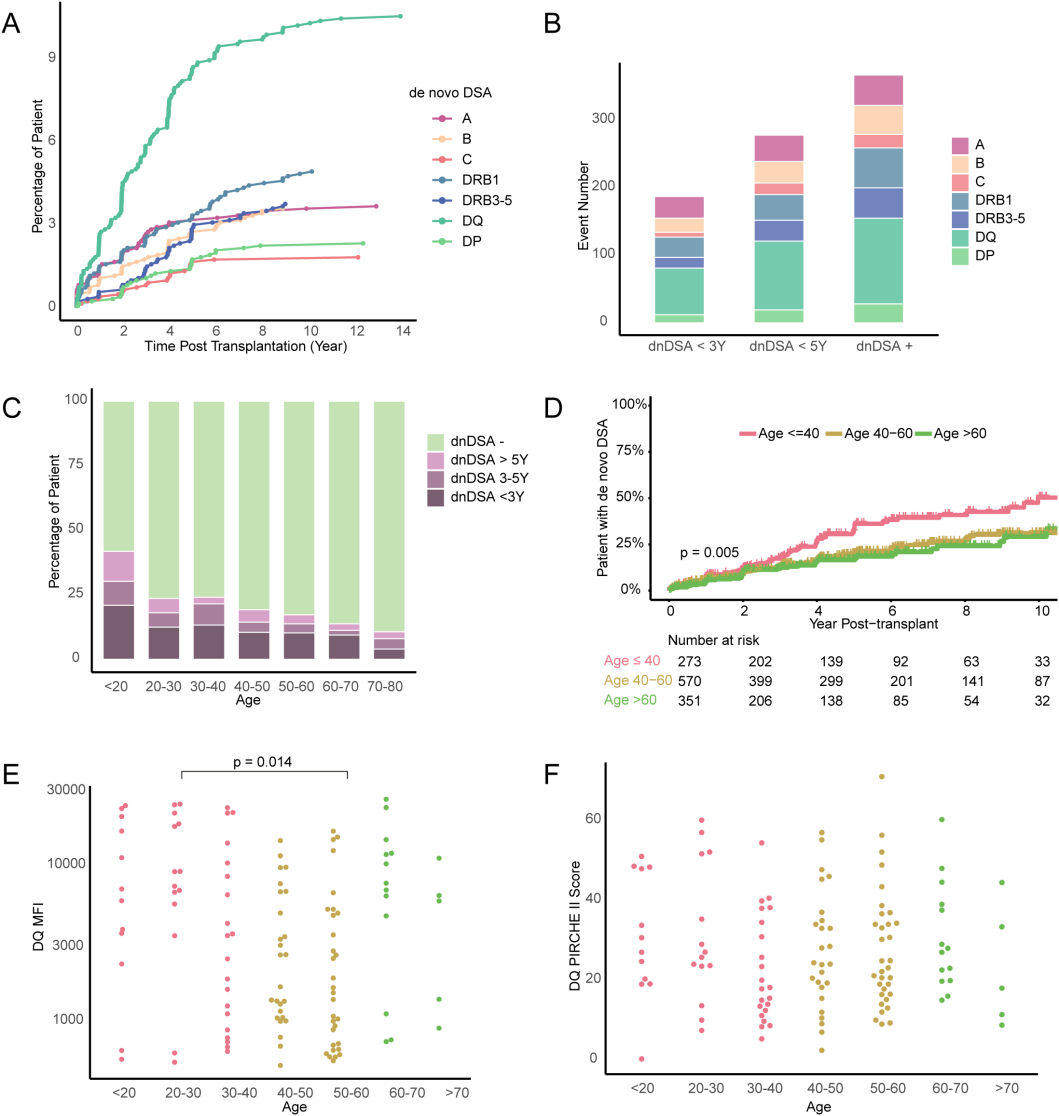
The relationship between the incidence of *de novo* DSA and age. **(A)** Line graph summarizing the frequency over time of patients developing single *de novo* DSA against HLA-A, HLA-B, HLA-C, HLA-DRB1 and HLA-DQ after transplantation. **(B)** Number of dnDSA occurrences grouped according to the time of dnDSA development post-transplant, the dnDSA + group contaings all dnDSA events recorded during the study period. **(C)** Percentage of patients with dnDSA development marked according to time after transplantation and grouped on age at transplantation. **(D)** Kaplan-Meier plots Indicating the cumulative incidence of *de novo* DSA stratified by age at transplantation (≤ 40, 40-60, >60). **(E)** MFI value of DQ dnDSA and **(F)** DQ PIRCHE II score in the different age groups.

Since we found differences in the kinetics of dnDSA development after transplantation in our different risk groups, we decided to investigate this further. We first compared the kinetics of dnDSA development against HLA-A, B, C, DR (including DRB3-5), DQ and DP within our cohort (**Figure 6A**). In this analysis, dnDSA development showed a similar kinetics against all loci with a more rapid increase in the first 2.5 years after transplantation, and followed by a tendency towards a flatter trajectory. Interestingly, dnDSA developed against HLA-DQ showed a trend towards a later flattening of the curve (**Figure 6A**). In order to compare the timing of dnDSA development stratified on target HLA loci, we compared dnDSA that developed within <3 years after transplant with those developing within <5 years after transplant, as well as the total number of dnDSA events (**Figure 6B**). In this analysis, we could not find any large differences, and instead, the relative composition of target HLA loci remained similar throughout the follow-up. We then investigated whether recipient age influenced the kinetics of dnDSA development. To do this, we examined the time of dnDSA development, stratified on different age groups (**Figure 6C**). In this analysis, we observed a clear difference in the fraction of patients within different age groups who developed dnDSA, indicating a trend towards more rapid dnDSA development in younger recipients (**Figure 6C**). Furthermore, hazard ratios from the Cox regression univariate analysis significantly exceeded 1.0 in the age group under 20 when compared to the mean age group (50-60, mean age 50.88) (**Table 2**). In a subsequent Kaplan Meier analysis, we observed a significantly higher dnDSA development in recipients who were <40 years old at the time of transplantation (**Figure 6D**). To investigate whether recipient age also impacted on dnDSA peak MFI, we again focused on dnDSA against HLA-DQ, our largest subgroup. We found significantly higher peak MFI values in recipients aged 20-30 years old compared to those who were 50-60 years old at the time of transplantation (**Figure 6E**). However, this difference did not appear to be associated with differences in the HLA-DQ PIRCHE II score between our age groups (**Figure 6F**).

**Table 2:** Univariate cox regression analysis for age groups on the prediction of dnDSA (n = 1194).

Age group univariate analysis		
	HR (95% CI)	P
<20	2.26 (1.30-3.92)	0.004
20-30	1.65 (0.95-2.86)	0.074
30-40	1.54 (1.00-2.38)	0.052
40-50	1.11 (0.73-1.69)	0.626
60-70	0.92 (0.59-1.43)	0.721
>70	1.05 (0.47-2.32)	0.910

CI, confidence interval; HR, hazard ratio; PIRCHE, predicted indirectly recognizable HLA epitopes.

Each age group compared to the mean age group (50-60).

## Discussion

The pre-transplant immunogenetical mismatch in the form of HLA mismatches between recipient and donor in the setting of kidney transplantation has been shown to have a significant impact on transplant outcome (7). This mismatch not only generates suitable targets for an alloimmune antibody response directed against the foreign HLA protein variants, but also exposes the recipient’s immune system to foreign HLA derived peptides. These allo-peptides then serve as the basis for this T cell dependent allo-immune response.

The PIRCHE II score aims to quantify the risk associated with an individual HLA locus mismatch by counting the number of possible immunogenic peptides that could potentially be presented by the recipient’s Class II molecules (8). A high score thus suggests that the recipient’s immune system will more easily find suitable targets for the alloimmune response, dependent on the increased likelihood that recipient T cells with TCR specificity for predicted allo HLA-derived peptides, encounter their cognate antigens. In our study, we found a significant association between a high PIRCHE II score and the development of dnDSA after kidney transplantation. This was particularly evident when we examined the risk associated with individual HLA loci PIRCHE II scores and the subsequent development of dnDSA targeting these individual locus mismatches.

The monitoring of anti-HLA antibody responses after transplantation using SAB technology is associated with several methodological challenges, where the validity of possible detected dnDSA must be evaluated in an individualized setting (13, 14). The annotation of false positive SAB beads as dnDSA might negatively impair our possibility to identify risk factors for dnDSA development. To address this, we employed a strategy of extended HLA typing and identification of unspecific patterns to categorize the detected dnDSA within our study. Interestingly, only dnDSA that were evaluated as true or possible were associated with an increased PIRCHE II score, further underlining the ability of this risk score to predict true dnDSA formation after transplantation.

We identified a significant association of the individual PIRCHE II score for all investigated HLA loci except HLA-C. This could be related to the relatively uncommon occurrence of true and possible dnDSA directed against HLA-C within our cohort (~6% of dnDSA), making it more challenging to detect such differences. We additionally found an association between the locus PIRCHE II score and dnDSA MFI, indicating that the PIRCHE II score could also be associated with the magnitude of the alloimmune response. In our cohort, HLA-DQ was the most common target for the detected dnDSA, which is in line with previous studies (15, 16). This is particularly problematic as prior studies have also shown that both pre-transplant and dnDSA directed against HLA-DQ are associated with a poor clinical outcome (4, 17). Our investigation into the impact of the total PIRCHE II score, compared to the single locus score, in the context of dnDSA directed against HLA-DQ suggests that the locus score is the primary predictor of risk. In contrast, the total score, excluding HLA-DQ PIRCHE II score, appears to have minimal influence on locus-specific dnDSA development.

The differences between the high and low quartile in the one locus HLA mismatch analysis also highlights the benefit of using the PIRCHE II score as opposed to relying on the classical mismatch count. Recipient age at the time of transplantation was also a clear risk factor for dnDSA development within our study. This could be related to the well-documented compliance problems associated with adolescence, as non-adherence with immunosuppressive therapy has been clearly shown to be a risk factor for dnDSA, rejection, and graft loss (18, 19). Our finding highlights the importance of the immunogenetical mismatch as a risk factor in a setting where the immunosuppression may be suboptimal. The increased risk of dnDSA formation in younger recipients could also be related to a potentially relatively diminished capacity for mounting strong allo-responses associated with an aging immune system (20). This was also evident in our older recipients, where dnDSA development was markedly diminished.

However, there are some limitations to our study. First, rates of antibody-mediated rejection (ABMR) were not analyzed in this study due to unavailable data; future studies should include ABMR data to strengthen these findings. Additionally, the use of imputed low-resolution typing for our PIRCHE-II calculations, rather than high-resolution typing, may limit the accuracy of immunogenetic risk assessment.

Taken together, our data suggest that pre-transplant PIRCHE II scores are important risk factors for the development of dnDSA, and that this is especially pronounced in younger transplant recipients. Given the strong association between PIRCHE-II scores and dnDSA development, particularly at the HLA-DQ locus, routine monitoring of PIRCHE-II scores could help identify patients at higher immunological risk, allowing for closer surveillance and potentially tailored immunosuppressive strategies to mitigate dnDSA-mediated graft injury (19, 21). Incorporating PIRCHE II scores into organ allocation algorithms, with a focus on younger recipients, could thus be an excellent strategy to mitigate dnDSA development and improve long-term transplant outcomes.

## Data Availability

The original contributions presented in the study are included in the article/**Supplementary Material**. Further inquiries can be directed to the corresponding author.

## Supplementary material

Supplementary Figure 1 Detail of *de novo* DSA target loci distribution. **(A)** Percentage of de dnDSA in patients with one dnDSA.

Supplementary Table 1 Number of *de novo* DSA combination in patients with multiple DSA.
