# Supplementary figures and images for: De novo donor-specific HLA antibody development after kidney transplantation is impacted by PIRCHE II score and recipient age
Source: Front Immunol. 2025 Apr 1;16:1508586. doi: 10.3389/fimmu.2025.1508586 (PMC11997444; doi:10.3389/fimmu.2025.1508586)

## Supplementary figure 1

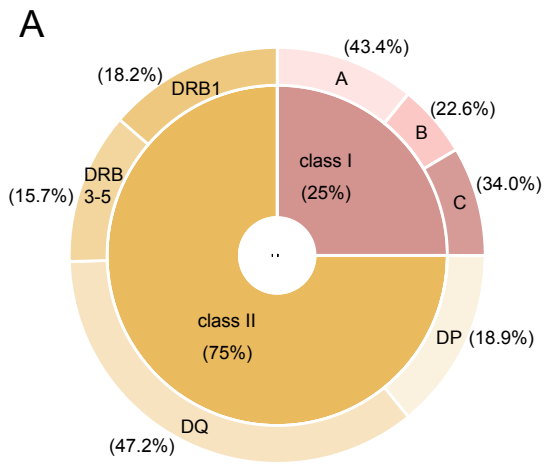

Supplement: Supplementary Figure 1 — Detail of de novo DSA target loci distribution. (A) Percentage of de dnDSA in patients with one dnDSA. [file DataSheet1.pdf]
