# Supplementary material for: De novo donor-specific HLA antibody development after kidney transplantation is impacted by PIRCHE II score and recipient age
Source: Front Immunol. 2025 Apr 1;16:1508586. doi: 10.3389/fimmu.2025.1508586 (PMC11997444; doi:10.3389/fimmu.2025.1508586)
Supplement: Supplementary Table 1 — Number of de novo DSA combination in patients with multiple DSA. [file Table1.docx]

| Supplementary Table 1. Number of de novo DSA combination in patients with multiple DSA | | | | |
| --- | --- | --- | --- | --- |
| de novo DSA | class I+I | class I+II | | class II+II |
| A, B | 6 |  |  | |
| A, B, C | 1 |  |  | |
| A, B, C, DP |  | 1 |  | |
| A, B, C, DQB |  | 2 |  | |
| A, B, C, DRB1, DP |  | 1 |  | |
| A, B, C, DRB1, DRB3-5, DQB, DP |  | 1 |  | |
| A, B, DQB |  | 3 |  | |
| A, B, DR3-5, DQB |  | 2 |  | |
| A, B, DRB1 |  | 1 |  | |
| A, B, DRB1, DQB |  | 4 |  | |
| A, B, DRB1, DQB, DP |  | 2 |  | |
| A, B, DRB1, DRB3-5 |  | 1 |  | |
| A, C, DP |  | 1 |  | |
| A, DQB |  | 1 |  | |
| A, DRB3-5, DP |  | 1 |  | |
| A, DR3-5, DQB |  | 2 |  | |
| A, DRB1 |  | 1 |  | |
| A, DRB1, DQB |  | 2 |  | |
| B, C, DQB |  | 1 |  | |
| B, DQB |  | 4 |  | |
| B, DRB3-5 |  | 1 |  | |
| B, DRB3-5, DQB |  | 2 |  | |
| B, DRB3-5, DQB, DP |  | 1 |  | |
| B, DRB1 |  | 2 |  | |
| B, DRB1, DQB |  | 2 |  | |
| B, DRB1, DRB3-5, DQB |  | 2 |  | |
| B, DRB1, DRB3-5, DQB, DP |  | 1 |  | |
| C, DP |  | 1 |  | |
| C, DQB |  | 3 |  | |
| C, DRB3-5 |  | 1 |  | |
| C, DRB1, DRB3-5, DQB |  | 1 |  | |
| DQB, DP |  |  | 4 | |
| DR5B3-5, DP |  |  | 2 | |
| DRB3-5, DQB |  |  | 11 | |
| DRB1, DP |  |  | 1 | |
| DRB1, DQB |  |  | 18 | |
| DRB1, DQB, DP |  |  | 4 | |
| DRB1, DRB3-5 |  |  | 8 | |
| DRB1, DRB3-5, DQB |  |  | 7 | |
| DRB1, DRB3-5, DQB, DP |  |  | 1 | |
